# Exposure to lipopolysaccharide (LPS) reduces contractile response of small airways from *GSTCD*^-/-^ mice

**DOI:** 10.1371/journal.pone.0221899

**Published:** 2019-09-12

**Authors:** Bo Liu, Amanda P. Henry, Sheyda Azimi, Suzanne Miller, Frank K. Lee, Jane C. Lee, Kelly Probert, Michael I. Kotlikoff, Ian Sayers, Ian P. Hall

**Affiliations:** 1 Division of Respiratory Medicine, & National Institute for Health Medicine, Nottingham Biomedical Research Centre, University of Nottingham, Nottingham, England, United Kingdom; 2 Department of Biomedical Sciences, College of Veterinary Medicine, Cornell University, Ithaca, New York, United States of America; Mohammed Bin Rashid University of Medicine and Health Sciences, UNITED ARAB EMIRATES

## Abstract

**Introduction:**

Genome-Wide Association Studies suggest glutathione S transferase C terminal domain (GSTCD) may play a role in development of Chronic Obstructive Pulmonary Disease. We aimed to define the potential role of GSTCD in airway inflammation and contraction using precision cut lung slice (PCLS) from wild-type (*GSTCD*^+/+^) and *GSTCD* knockout mice (*GSTCD*^-/-^).

**Methods:**

PCLS from age and gender matched *GSTCD*^+/+^ and *GSTCD*^-/-^ mice were prepared using a microtome. Contraction was studied after applying either a single dose of Methacholine (Mch) (1 μM) or different doses of Mch (0.001 to 100 μM). Each slice was then treated with lipopolysaccharide (LPS) or vehicle (PBS) for 24 hours. PCLS contraction in the same airway was repeated before and after stimulation. Levels of TNFα production was also measured.

**Results:**

There were no differences in contraction of PCLS from *GSTCD*^+/+^ and *GSTCD*^-/-^ mice in response to Mch (EC_50_ of *GSTCD*^+/+^ vs *GSTCD*^-/-^ animals: 100.0±20.7 vs 107.7±24.5 nM, p = 0.855, n = 6 animals/group). However, after LPS treatment, there was a 31.6% reduction in contraction in the *GSTCD*^-/-^ group (p = 0.023, n = 6 animals). There was no significant difference between PBS and LPS treatment groups in *GSTCD*^+/+^ animals. We observed a significant increase in TNFα production induced by LPS in *GSTCD*^-/-^ lung slices compared to the *GSTCD*^+/+^ LPS treated slices.

**Conclusion:**

*GSTCD* knockout mice showed an increased responsiveness to LPS (as determined by TNFα production) that was accompanied by a reduced contraction of small airways in PCLS. These data highlight an unrecognised potential function of GSTCD in mediating inflammatory signals that affect airway responses.

## Introduction

Genome-wide association study meta-analyses have identified associations between genetic variants located on Chromosome 4q24 and lung function parameters including forced expiratory volume in 1 second (FEV_1_), the ratio of FEV_1_ to forced vital capacity (FEV_1_/FVC)), as well as Chronic Obstructive Pulmonary Disease [1–6]. Several genes sit at this locus, and the credible variant set includes variants in the glutathione S-transferase C-terminal containing domain (*GSTCD*) gene [4, 5, 7, 8]. Additional evidence supporting a potential role for this gene comes from expression quantitative trait loci (eQTL) data which show that increased *GSTCD* mRNA levels correlate with better lung function [8, 9]. GST genotypes have also been shown to be associated with the degree of hyper-responsiveness in adults with asthma [10].

GSTCD has a structural domain related to GSTs which themselves consist of three super families defined according to location (cytosol, mitochondrial and microsomal). Cytosolic GSTs possess more than 40% sequence homology and consist of 13 different classes based on their structure [7]. GST is best known for its enzymatic function in detoxification, breaking down the reduced form of glutathione contained in some toxins to xenobiotic substrates. GST can also function as a transport protein and has been shown to be involved in signal transduction pathways that control cell proliferation and death [11–14]. It is not known if GSTCD also possesses enzymatic activity in the respiratory system.

mRNA and protein expression studies in airway and lung tissue suggest that expression of GSTCD is ubiquitous in lung tissue [8]. It is found to be expressed most strongly in human bronchial epithelial cells (HBECs), both at the mRNA and protein level [8]. In addition, GSTCD protein expression was observed to be highest in the earlier stages of foetal development [15].

In this study, two different *GSTCD* knock-out mouse models were used to investigate phenotype correlations and specifically to look at methacholine (Mch)-induced contraction of small airways. This was examined in both wild-type (*GSTCD*^+/+^) and *GSTCD* knock-out (*GSTCD*^-/-^) mice using the precision cut lung slice (PCLS) technique to determine whether there are any changes in contractility of airways in *GSTCD*^-/-^ mice. In addition, we studied the effect of a pro-inflammatory stimulus on these responses using pre-incubation with bacterial endotoxin LPS, which is associated with airway inflammation in diseases such as COPD [16–19].

## Materials and methods

### Animal work

All animal work was carried out under humane conditions, approved by the Cornell Institutional Animal Care and Use Committee (USA) and adhered to the standards set out in the *Guide for the Care and Use of Laboratory Animals*, *8*^*th*^ *edition 2011*. Work in the UK was performed under Project licences PPL 40/3576 and P57452337.

Details of *GSTCD*^-/-^ animals used for the work described in this paper can be found in the online (S1 Text) supporting information (See also S1 Fig and S2 Fig). Breeding and genotyping of the age and sex matched animals is also described in supporting information.

### Study of contraction of small airways by use of PCLS

#### Preparation of mice lung slices

PCLS were prepared as previously described [20–22]. Briefly, age, sex, litter mate matched mice (one *GSTCD*^+/+^ mouse and one *GSTCD*^-/-^ mouse) were sacrificed (aged between 50 and 57 days) by peritoneal injection of pentobarbital (0.3 ml per mouse). After confirmation of death, the lung and bronchus were exposed. A 20G mice bronchial tube was inserted and pre-prepared 2% low-temperature (1.2 ml) agarose was injected slowly through the tube. A final bolus of air (0.2 ml) was injected to push the agarose into the small airways and alveoli. The agarose in the lung was jellified by applying ice cold cotton over the lung for about 20 minutes. The lung was then put on ice to allow the agarose to set. Lobes were dissected in a petri dish filled with cold HBSS (Invitrogen) containing HEPES (20 mM) pH 7.4, then 20 slices each 150 μM thick were cut from the left lobe of each animal using an OTS-5000 microtome machine (Electron Microscopic Sciences, PA, and USA). The slices were incubated at 37°C for an hour in an incubator gassed with 5% CO_2_ and then washed in DMEM (Gibco) 3 times to remove agarose followed by an overnight incubation prior to contraction studies being performed.

### Microscopic study of contraction of lung slices

PCLS were held in place by a slice anchor (Harvard Apparatus, UK) in a perfusion chamber mounted on the platform of an Inverted microscope (Diaphot 300, Nikon, Japan). A range of concentrations of Mch (Sigma) dissolved in HBSS/HEPES buffer was delivered at a rate of 5ml per minute through an 8-channel perfusion system using a computer-controlled programme (EasyCode®, Automate Scientific): 0.4ml volumes surrounded the slice throughout the study. PCLS with intact structures and beating cilia were selected for contraction studies. Either single dose studies (three slices from each animal) or dose response studies (two slices from each animal) were performed. In the single dose study, a submaximal dose of Mch (1 μM) was perfused to the slice for five minutes after equilibration in HBSS/HEPES buffer at room temperature for 5 minutes. For dose response studies, different doses of Mch (0.001 to 100 μM) were delivered via different channels of the perfusion system (5 minutes for each dose) followed by a 10 minute wash with HBSS/HEPES buffer. Bright field images were taken by CCD camera on a microscope (Nikon Diaphot 300, Nikon) at a frame rate of 1 per 5 second using the SPOT programme (SPOT Imaging, Spot Advance Software, USA). Cross-section areas of bronchi were measured by pixel summing using image analysis software (ImageJ) [23, 24]. An averaged contraction from the last one minute of each perfusion period was calculated and thus the mean response calculated for each animal. Comparisons between *GSTCD*^+/+^ and *GSTCD*^-/-^ mice were made. Experiments were conducted with the investigator blind to genotype.

### Incubation of PCLS with LPS

The effect of LPS from *Escherichia coli* O26:B6 (Sigma L2654) on contraction of PCLS was studied using both the single dose and dose-response protocols. Immediately after an initial contraction study, the slice was transferred into chambers containing HBSS/HEPES buffer with either LPS (10 μg/ml) or PBS and incubated for 24 hours. This concentration of LPS was selected based on a previous study on murine lung slices [18]. Contractile responses were studied post PBS or LPS stimulation. Differences in contractile responses before and after treatment were compared between *GSTCD*^+/+^ and *GSTCD*^-/-^ mice.

### Determination of TNFα production following incubation of PCLS with LPS

Following 24 hours of incubation of PCLS with LPS, supernatant’s were collected and stored at -80°C. TNFα is a pro-inflammatory mediator known to be induced by LPS in the lung [18, 25–28]. The concentration of TNFα was measured using a murine TNFα Elisa kit (DY410-05, Duoset; R&D system Minneapolis, USA) as directed by the manufacturer. The levels of TNFα were compared among the different groups (6 slices in each group).

### Statistics

Paired Student T-tests were performed in excel to compare the difference between groups. P ≤ 0.05 was considered significant.

## Results

### GSTCD expression in *GSTCD*^+/+^ mice

**Fig 1:**
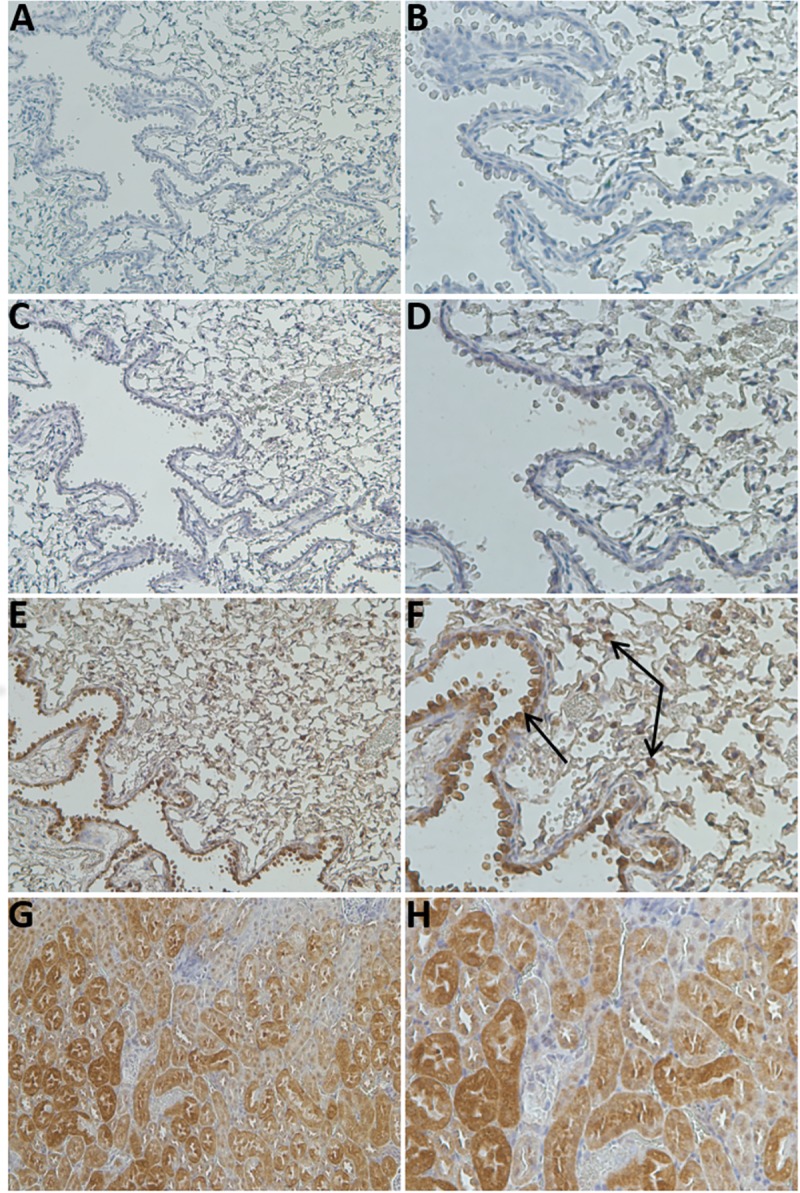
GSTCD protein expression indicates bronchial epithelial localisation as assessed by IHC in *GSTCD^+/+^* C57BL/6N mouse lung tissue. Representative images of GSTCD expression with negative and isotype controls in lung tissue and positive control performed in kidney tissue. No staining is evident in either negative control (A-D). Predominant GSTCD staining in the lung indicates bronchial epithelial specificity in lung tissue and specific cells across the alveolar tissue (E, F, arrows indicate localised staining) and predicted positive control staining is seen in kidney tissue (G, H). Sections were stained with Proteintech antibody at 1:50 and images shown at x20 (A, C, E, G) and x40 (B, D, F, H) magnification. Analysis was performed on sections of paraffin embedded adult lung tissue age matched at 10 weeks. See also S2 Fig.

GSTCD expression was initially studied by immunohistochemistry (IHC) in GCTCD+/+ mouse lungs (Fig 1).

### Phenotype of *GSTCD*^-/-^ mice

**Fig 2:**
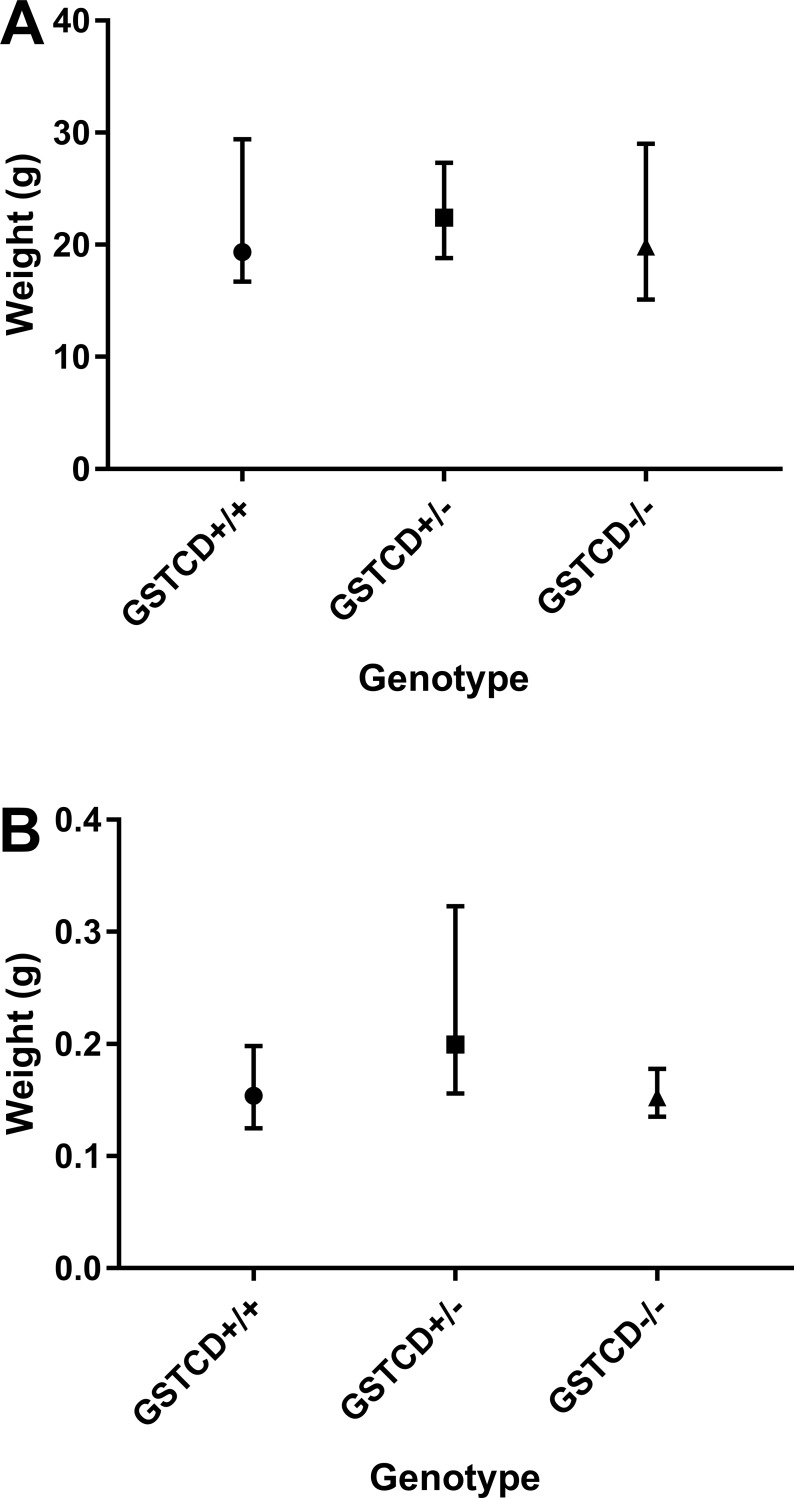
GSTCD deletion does not affect total mouse or lung wet weight. Whole body (**A**) and wet lung (**B**) median and range of weight in grams for 6 *GSTCD*^+/+^ mice, 6 Heterozygous *GSTCD*^*+*/-^ mice and 6 *GSTCD*^-/-^ mice.

*GSTCD*^-/-^ mice were viable and showed no significant change in total mouse or lung weights between the *GSTCD*^+/+^ or *GSTCD*^-/-^ animals (Fig 2).

### GSTCD and LacZ staining in *GSTCD*^+/+^ and *GSTCD*^-/-^ mice

Positive anti-GSTCD antibody staining was observed in the +/+ mice (Fig 1 and S2 Fig), no staining was observed in the -/- mice (S2 Fig). *GSTCD*^-/-^ mice showed strongest LacZ staining in the airways showing that GSTCD was knocked out (also shown in S2 Fig).

### Non-respiratory phenotypes of *GSTCD*^-/-^ mice

Data were interrogated from http://www.mousephenotype.org/data/genes/MGI:1914803#section-associations Phenotype abnormalities described in the IMPC *GSTCD*^-/-^ mice include; increased or absent threshold for auditory brainstem response (p = 5x10^-6^), decreased mean corpuscular haemoglobin concentration (p = 4.55x10^-5^), decreased circulating alanine transaminase level (p = 9.5x10^-5^) and decreased startle reflex in both male and females (p = 0.0002) as well as increased spleen weight in males (one out of 13 animals) and decreased lean body mass in females (p = 1.49x10^-5^), all compared to the *GSTCD*^+/+^ animals. No baseline respiratory abnormalities were recorded.

### Comparison of contraction between *GSTCD*^+/+^ and *GSTCD*^-/-^ mice

**Fig 3:**
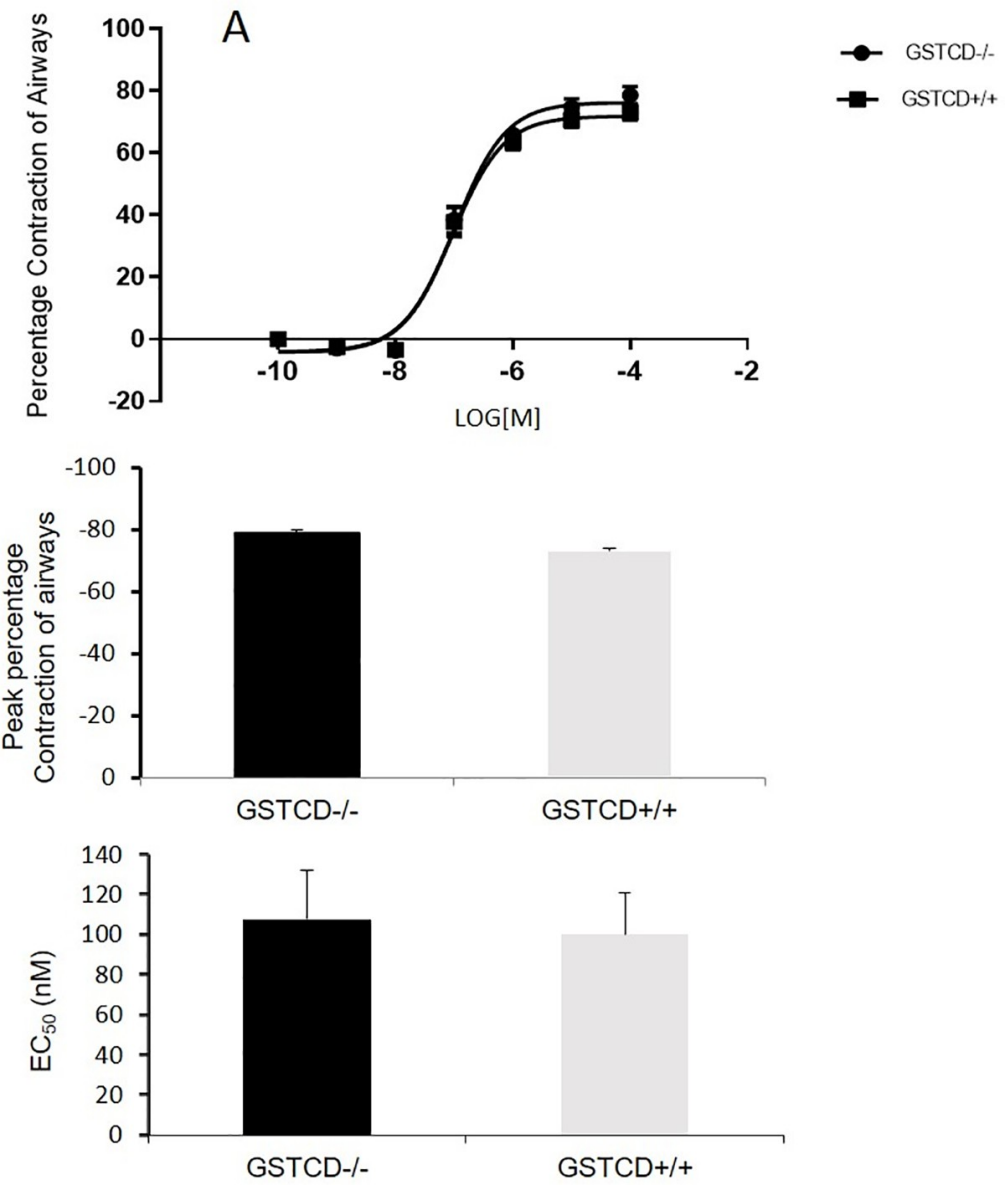
GSTCD deletion does not influence Methacholine induced contraction in PCLS. Peak percentage contraction of airways (**A**) comparison of dose-response curves (**B**), and EC_50_ (**C**) between *GSTCD*^+/+^ and *GSTCD*^-/-^ mice. Dose-response curves were created by applying different concentration of Mch (0.001 to 100 μM), each concentration for 5 min and averaged contraction during the final minute of each addition was calculated. Data are means from n = 6 animals in each group; 2–3 slices were studied from each animal.

Contractility of mice airways was compared between *GSTCD*^-/-^ mice and *GSTCD*^+/+^ age and sex matched litter mate mice. Lung slices with epithelial integrity proven by cilia beating and a good test response to Mch were subjected to either single doses of Mch (1 μM, 3 slices for each mouse) or dose dependent responses (0.001–100μM, 2 slices for each mouse). We observed no differences in either maximum contractility to a single dose 1μM of Mch (Fig 3A) or in dose-response curves between *GSTCD*^-/-^ mice and *GSTCD*^+/+^ mice (Fig 3B) and EC_50_ (Fig 3C) (both p<0.05, n = 12–18 slices from 6 animals per group).

### Comparison of contractility of airways following LPS treatment

**Fig 4:**
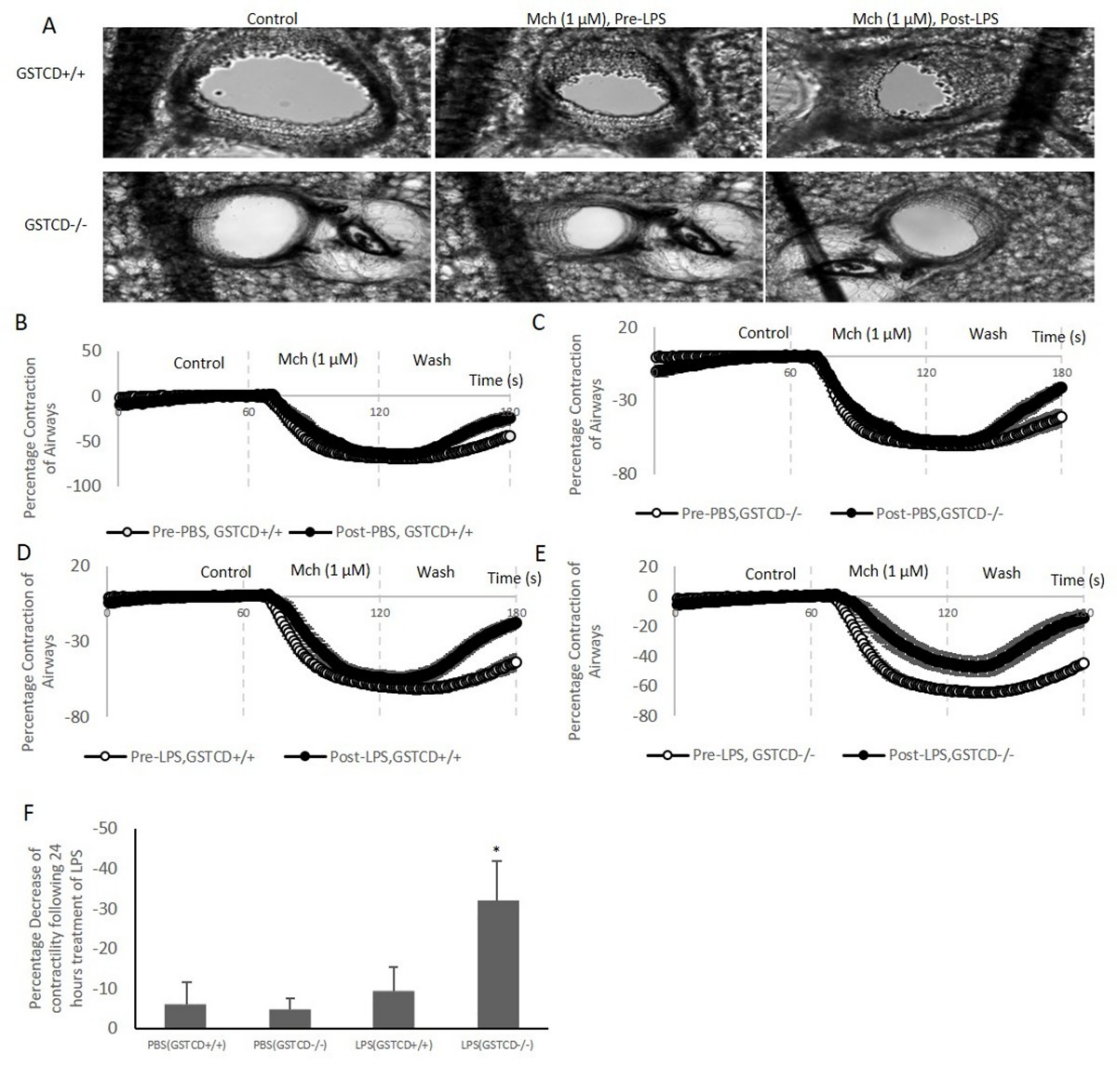
LPS treatment leads to contractile responses only in the GSTCD deleted PCLS. Lung slices were prepared from *GSTCD*^+/+^ and *GSTCD*^-/-^ mice. (**A**) And (**B**) comparison of time courses of averaged contraction of slices in *GSTCD*^+/+^ following either PBS or LPS treatment. (**C**) And (**D**) comparison of time courses of averaged contraction of slices in *GSTCD*^-/-^ following either PBS or LPS treatment. (**E**) Representative airway in *GSTCD*^+/+^ (upper row) and *GSTCD*^-/-^ (lower row) stimulated by Mch (1 μM) before and after LPS treatment. Images of airways were taken following a 5-minute incubation of drugs and control (in absence of Mch). (**F**) Comparison of % decrease of contractility of airways before and after LPS treatment in *GSTCD*^+/+^ and *GSTCD*^-/-^ groups. * Compared between LPS in *GSTCD*^+/+^ PCLS and *GSTCD*^-/-^ PCLS *P* <0.05.

**Fig 5:**
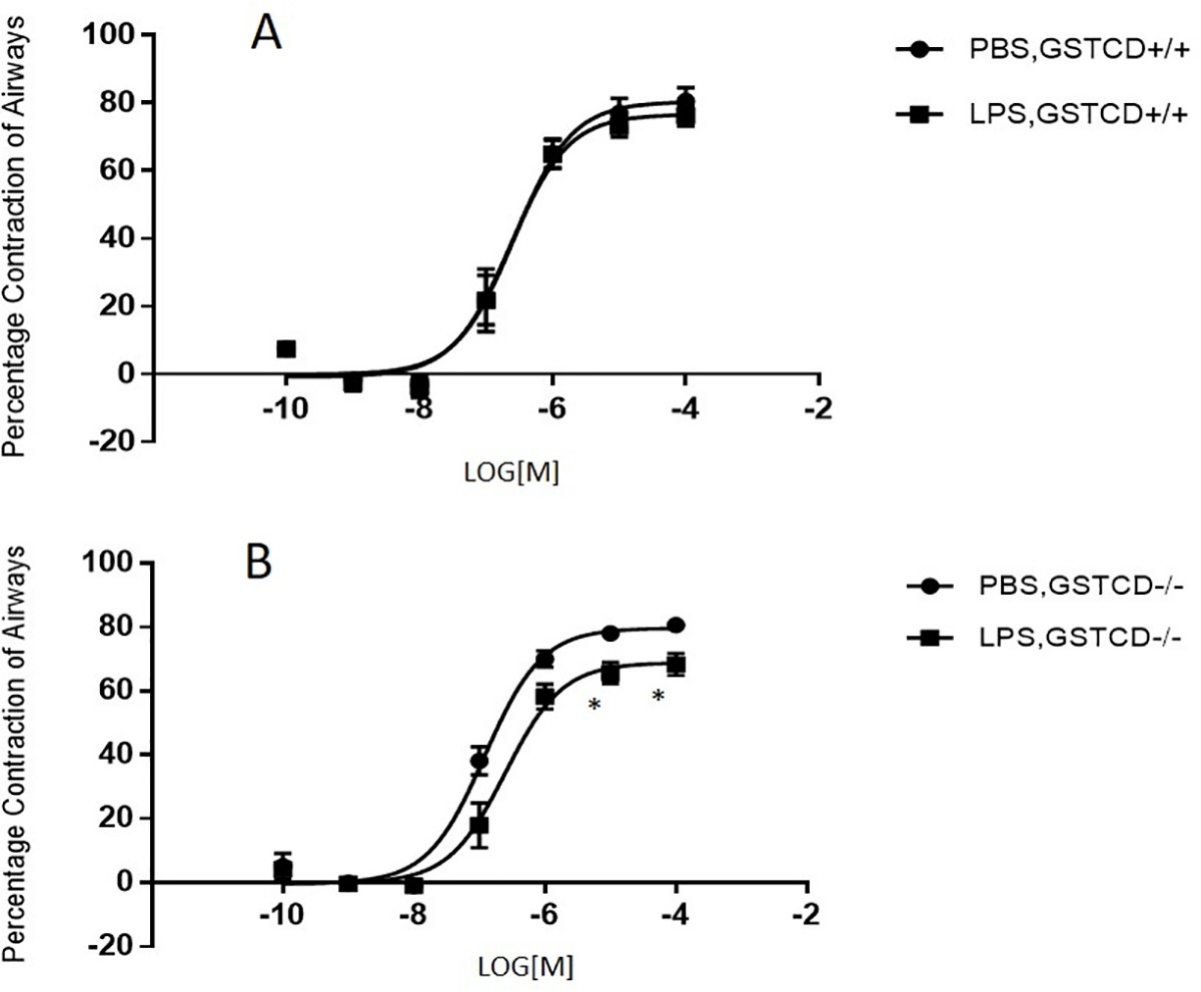
Dose response of airways to Methacholine shows a decreased contractility in *GSTCD*^-/-^ mouse lungs following LPS treatment. Dose-response curves were produced (**A**) *GSTCD*^+/+^ (**B**) *GSTCD*^-/-^ by applying different doses of Mch (0.001 to 100 μM) to the slices for five minutes. Percentage contraction of airways during the last minute from 2 or 3 slices each animal was averaged and a total of 6 animals in each group were compared. In the *GSTCD*^-/-^ group dose-response curves were right-shifted. *P <0.05 compared with the same concentration following LPS treatment.

Given the potential involvement of GSTCD homologues in inflammatory signalling we next examined contractility following LPS treatment of *GSTCD*^-/-^ and *GSTCD*^+/+^ lung slices. Initial experiments demonstrated that there was a small reduction (<10%) in contractile responses to a sub maximum dose of Mch (1uM) following 24 hours incubation of GSTCD^+/+^ PCLS in the absence of LPS, possibly related to background release of mediators from slices. In *GSTCD*^+/+^ mice (Fig 4A and 4B), this reduction appeared larger when LPS was included in the media during the 24h incubation period although this difference was not significant. However, there was increased reduction of contraction of airways in *GSTCD*^-/-^ mice (Fig 4C and 4D) following LPS pre-treatment in response to challenge with 1uM Mch. Fig 4D shows representative images of airways stimulated with Mch (1μM) before and after LPS challenge. The difference between PBS and LPS treated groups of slices was significant (4.7% vs 32.8%, p<0.05, slices from n = 6 animals) (Fig 4F). There was no shift in the GSTCD^+/+^ PCLS dose-response curves to Mch (Fig 5A), however a rightwards shift in PCLS from the LPS-treated group of *GSTCD*^-/-^ mice was observed (Fig 5B). EC_50_ values were not significantly different as follow: PBS-treated 123±27nM (*GSTCD*^+/+^) and 135±54nM (*GSTCD*^-/-^); LPS-treated 139±34nM (*GSTCD*^+/+^) and 258±76nM (*GSTCD*^-/-^), all n = 6, p>0.05.

### LPS treatment induces higher TNFα production in PCLS *GSTCD*^-/-^ mice

**Fig 6:**
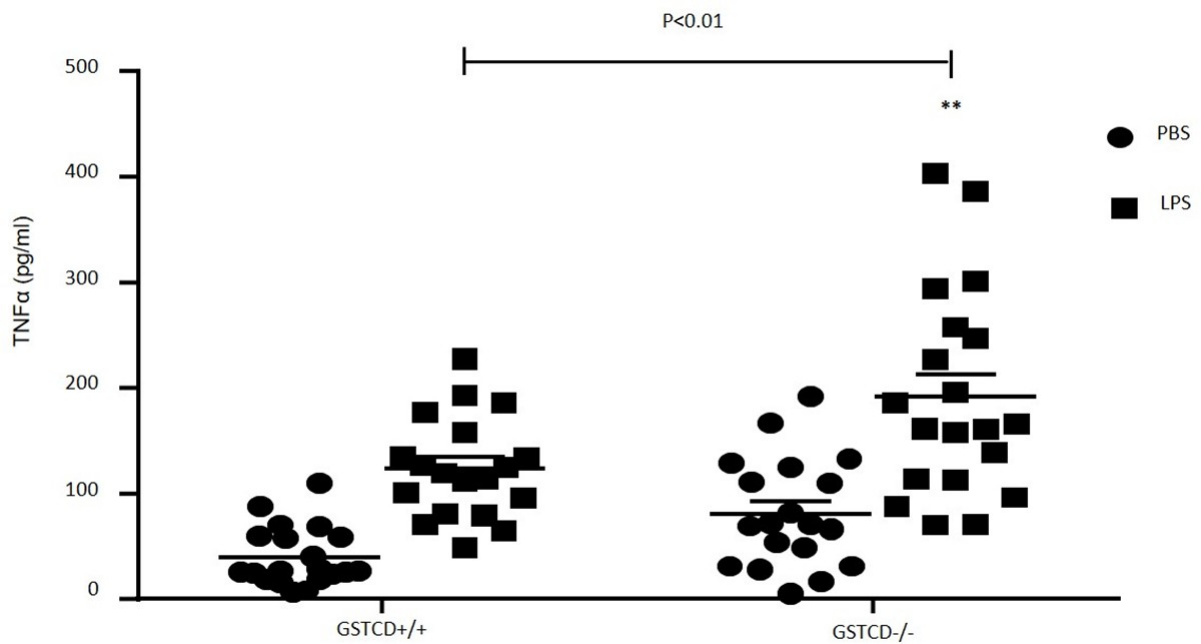
TNFα release from *GSTCD*^+/+^ and *GSTCD*^-/-^ airways before or after LPS (10 μg/ml) treatment. Supernatants following either PBS or LPS treatment were collected and concentration of TNFα (pg/ml) was measured using ELISA. *P<0.01, n = 18–20 supernatants from 6 mice.

To further assess possible mechanisms underlying the effect of *GSTCD* genotype on LPS pre-treatment on contractile signalling, we studied the release of TNFα, a pro-inflammatory mediator known to be induced by LPS in the lung. Following 24h LPS treatment, there was a marked increase of TNFα release in slices from both *GSTCD*^+/+^ mice and *GSTCD*^-/-^ mice in comparison to the PBS treated group (Fig 6). However, there was a greater increase of TNFα production in PCLS from *GSTCD*^-/-^ when compared to *GSTCD*^+/+^ mice following LPS treatment. Basal levels of TNFα release also appeared higher in the *GSTCD*^-/-^ slices, although this difference was not statistically significant. In order to further study the possible contribution of TNFα to the decreased contraction of airway in *GSTCD* knockout mice, we compared the percentage decrease of contractility of airways which were subjected to 24 hours of treatment of TNFα between *GSTCD*^+/+^ and *GSTCD*^-/-^ mice. Incubation with TNFα (10 ng/ml) showed no effects on contraction of airways in *GSTCD*^+/+^ mice. However, there was a small decrease in contractility of airways both in untreated and following 24 hours of TNFα treatment in *GSTCD*^-/-^ mice (38±6%, n = 6) suggesting TNFα may at least partially mediate the decreased contractility of airways seen in *GSTCD*^-/-^ mice after LPS stimulation, although this difference was not significant.

## Discussion

In this study, we set out to try and explore the potential role of GSTCD in the control of airway responses using PCLS from transgenic mouse models. Given genetic data implying a potential role for GSTCD in the control of lung function [10], gaining a clear understanding of the function of GSTCD would be valuable. To do this we compared the difference of contractility of small airways from *GSTCD*^-/-^ and *GSTCD*^+/+^ mice. Under non-inflammatory conditions, airways from *GSTCD*^-/-^ mice behaved similarly to airways from *GSTCD*^+/+^ mice. However, airways prepared from the lungs of *GSTCD*^-/-^ mice showed reduced contractility in an inflammatory environment created by incubation of PCLS with LPS, suggesting GSTCD may regulate LPS-induced airway inflammation and hence potentially airway contraction in patients under inflammatory conditions such as those in the context of infective exacerbations of asthma or COPD.

Using two different *GSTCD*^-/-^ models we also explored the phenotype of these animals in more detail. The mice are viable, and show no obvious differences in terms of breeding success or other major abnormalities. Data from IMPC show that *GSTCD*^-/-^ mice have increased spleen weight in males, decrease lean body mass in females, decreased circulating phosphate levels and decreased startle reflexes in both male and females compared to the *GSTCD*^+/+^ animals. Our data show no obvious differences in terms of lung development or overall body weight between *GSTCD*^-/-^ and *GSTCD*^+/+^ mice.

Currently the pathophysiological role of GSTCD in asthma or COPD is not fully understood. The genetic association studies and eQTL data show association between variants associated with increased mRNA for GSTCD and better lung function, and hence it is possible that genetic variants which alter GSTCD expression may underlie the associations seen in population studies. GSTCD is expressed in structural cells in the respiratory system including airway smooth muscle cells and bronchial epithelium [8, 29]. GSTCD has significant homology with other GST proteins which are important in controlling redox states in the lung [30, 31]. GSTM1 and GSTT1 have also found to be associated with control of airway hyper-responsiveness, and GSTP1 has been found to prevent LPS-induced inflammation and play an anti-inflammatory role in the response to LPS by inhibiting LPS-induced mitogen-activated protein kinases (MAPKs) [32, 33]. However, GSTCD lacks the key functional domains believed to be important for these activities. It therefore remains unclear if the association of GSTCD with lung function is connected with the enzymatic function possessed more generally by the GST family.

In addition, a protein homology search also revealed homology between GSTCD and chloride intracellular channels (CLICs) 1, 3, 4, 5 and 6, suggesting an alternative mechanism whereby GSTCD could contribute to control of airway contraction. A recent study from our group has demonstrated that CLICs play a role in Ca^2+^ homeostasis in the airways, with CLIC1 contributing to the modulation of cAMP-induced whole cell currents in human bronchial epithelial cells [34]. Hence it is possible that the effects of GSTCD on airways are mediated via altered Cl^-^ channel activity. In keeping with this suggestion it has been shown that some other GSTs can also modulate CLIC channel activity [30, 31, 35].

In the current study we found that incubation of lung slices with LPS in *GSTCD*^-/-^ reduced contractility compared with the response seen in *GSTCD*^+/+^ mice Interestingly, in *GSTCD*^+/+^ mice we found that pre-incubation with LPS did not alter small airway contractility in lung slices: these findings are in keeping with some previous observations [18]. The observation that contractility of airways was reduced in *GSTCD*^-/-^ mouse lung slices suggests that GSTCD may play a role in maintaining contractile signalling per se. This however does not explain the eQTL data which show variants associated with lower levels of GSTCD expression and better lung function.

To further investigate the potential role of GSTCD in control of inflammatory signalling in the airways we went on to study TNFα production in lung slices. LPS binds to and activates Toll-like receptor 2 and 4 (TLR2 and TLR4) depending on the type of LPS which leads to activation of pro-inflammation pathways such as myeloid differentiation factor /NFκB, phosphoinositide 3-kinase/Akt and MAPKs [18, 36–39]. Activation of TH2 cells will also lead to release of other pro-inflammatory cytokines such as TNFα and IL-1β. In our study we found that following treatment with LPS there was a marked increase in TNFα release in both *GSTCD*^+/+^ and *GSTCD*^-/-^ mice. In addition, LPS induced TNFα release was significantly higher in PCLS from *GSTCD*^-/-^ mice, and although not significant there was a trend towards increased TNFα release in unstimulated PCLS as well. This shows that reduced contractility in GSTCD slices treated with LPS is associated with enhanced TNFα release. Therefore we examined the effect of direct TNFα application on lung slices: whilst we saw some reduction in contractility in PCLS from *GSTCD*^-/-^ mice after pre-incubation with TNFα this could not explain the whole of the difference in LPS induced responses seen between *GSTCD*^-/-^ and *GSTCD*^+/+^ PCLS suggesting additional mechanisms must also contribute. Interestingly, previous work has shown that contractile responses by themselves can regulate NFkB activation which can in turn lead to altered TNFα production providing a further way in which the degree of contraction can influence mediator release and subsequent responses [40]. In addition, there is evidence that TNFα may produce a reduced contractile response in PCLS (as opposed to isolated airway myocytes where enhanced Ca2+ responses are seen following TNFα pre-incubation) through regulation of NMDA-R mediated responses [41].

In summary, using PCLS, we have shown that *GSTCD*^-/-^ mice show an increased responsiveness to LPS (as determined by TNFα production) that was accompanied by a reduced contraction of small airways. These data potentially highlight an unrecognised function of GSTCD in mediating inflammatory signals that affect airway calibre, and may in part underlie the genetic associations seen at the GSTCD locus with lung function and COPD phenotypes.

## Supporting information

S1 Text Supporting information. Generation of the mouse model and methods used to genotype the animals.
(DOCX)

S1 Fig GSTCD knockout cassette and region on chromosome 4q24. (A) The Promoter driven cassette used to produce the knockout mouse and (B) the region of GSTCD sequences (5’ arm and 3’ arm) inserted into the cassette to make it gene specific on Chromosome 3 GRCm38.p1 C57BL/6J accession number NM_080507.
(JPG)

S2 Fig Immunohistochemical analysis of *GSTCD*^-/-^ mouse. The lung sections show control (no antibody) and stained with anti-GSTCD antibody respectively (**A and B**) show *GSTCD*^+/+^ and (**C and D**) *GSTCD*^*-/-*^ lungs. (**E**) Xgal staining in GSTCD^-/-^ lung for LacZ expression. Scale bar– 200 μm.
(JPG)
