# Supplementary material for: Exposure to lipopolysaccharide (LPS) reduces contractile response of small airways from GSTCD-/- mice
Source: PLoS One. 2019 Sep 12;14(9):e0221899. doi: 10.1371/journal.pone.0221899 (PMC6742219; doi:10.1371/journal.pone.0221899)
Supplement: S1 Text — Generation of the mouse model and methods used to genotype the animals. (DOCX) [file pone.0221899.s001.docx]

## **Supplementary methods**

# **Animal work**

All animal work was carried out under humane conditions, approved by the Cornell Institutional Animal Care and Use Committee (USA) and adhered to the standards set out in the Guide for the Care and Use of Laboratory Animals, 8^th^ edition 2011. Work in the UK was performed under Project licences PPL 40/3576 and P57452337.

**Animals**

The *GSTCD^-/-^* strain originated at Cornell University. C57BL/6N (black) embryonic stem cells carrying a targeted knock-out of GSTCD (Fig. S1) were injected into C57BL/62J (white) blastocysts. Chimeric offspring were mated to C57BL/62J and black pups indicated germline transmission (International Mouse Phenotyping Consortium (IMPC) – GSTCD http://www.mousephenotype.org/data/genes/MGI:1914803). Additional data became available during the time of this research from mice generated by the IMPC itself.

**Breeding GSTCD mice**

Once mice were received in Nottingham the breeding pairs were set up using heterozygous animals to obtain both *GSTCD*^+/+^ and *GSTCD*^-/-^ offspring from the same parents to give matched age and sex experimental animals.

**Genotyping GSTCD mice**

Once the pups reached day 23, ear notches were taken for genotyping and identification. The Phire Animal Tissue direct PCR Kit (Thermo Scientific Cat. No. F-140WH) was used to prepare samples for PCR and genotype analysis as follows. The frozen (-20^o^C) ear notch was placed in 20µl Dilution buffer, 0.5µl DNA Release Additive was added and mixed by vortexing briefly and centrifuging. This was then incubated at room temperature for 5 minutes before placing at 98^o^C for 2 minutes. The sample was then stored at -20^o^C until PCR was performed. Two 25µl PCR reactions were set up using 1µl of dilution sample in each, one was set up using the *GSTCD*^+/+^ primer pair and the second using the *GSTCD*^-/-^ (lacZ) primer pair. Sequences and PCR conditions are shown in Table S1. The resulting PCR products were then electrophoresed on a 1% agarose gel containing 0.5µg/ml Ethidium Bromide a band on the gel representing the presence of either the *GSTCD*^+/+^ or *GSTCD*^-/-^ allele.

**Table S1.** PCR primers, conditions and band sizes expected from the PCR for genotyping the GSTCD animals.

| Primer name | Sequence | PCR condition | Product size |
| --- | --- | --- | --- |
| GSTCD+/+ for | GCTGGAACAGTTTCCACTACTA | 57^o^C  30 cycles | 200bp |
| GSTCD+/+ rev | TCGGTCTTGGTCCTCCTATAA |  |  |
| lacZ for GSTCD-/- | CGATCGTAATACCCGAGTGT | 58^o^C  35 cycles | 800bp |
| lacZ rev GSTCD-/- | GCGGTACTTCAGGCAGTTCAA |  |  |

**Immunohistochemistry and X-gal staining**

For anti-GSTCD immunohistochemistry paraffin sections were prepared from lungs fixed in 4% PFA overnight at 2-8ºC. Following deparaffinization, endogenous peroxidase activity was treated with 3% hydrogen peroxide in methanol and non-specific binding was blocked with normal goat serum (Vector Laboratories). The blocked sections were incubated overnight at 2-8 ºC with rabbit anti-GFP polyclonal antibody (1/100 dilution, Santa Cruz sc-138332) and developed with biotinylated goat anti-rabbit secondary antibody/HRP-conjugated streptavidin/AEC reagent combination. The slides were counterstained with hematoxylin, mounted with Fluoromount G and digitally scanned on Aperio CS2 (Leica Biosystems). Sections from non-transgenic littermates were included as positive control (Fig. S2).

For X-gal staining, the PFA-fixed lung was dehydrated in 30% sucrose, mounted in OCT and cryosectioned. The cryosections were washed in 1x PBS and incubated overnight in X-gal developing solution. The sections were mounted with Fluoromount G and digitally scanned.

**Supplementary figure legends**

**Fig. S1: GSTCD knockout cassette and region on chromosome 4q24**. (A) The Promoter driven cassette used to produce the knockout mouse and (B) the region of GSTCD sequences (5’ arm and 3’ arm) inserted into the cassette to make it gene specific on Chromosome 3 GRCm38.p1 C57BL/6J accession number NM_080507.

**Fig. S2: Immunohistochemical analysis of *GSTCD*^-/-^ mouse.** The lung sections show control (no antibody) and stained with anti-GSTCD antibody respectively (**A and B**) show *GSTCD*^+/+^ and (**C and D**) *GSTCD^-/-^* lungs. (**E**) Xgal staining in GSTCD^-/-^ lung for LacZ expression. Scale bar – 200 µm.
