# Supplementary figures and images for: Exposure to lipopolysaccharide (LPS) reduces contractile response of small airways from GSTCD-/- mice
Source: PLoS One. 2019 Sep 12;14(9):e0221899. doi: 10.1371/journal.pone.0221899 (PMC6742219; doi:10.1371/journal.pone.0221899)

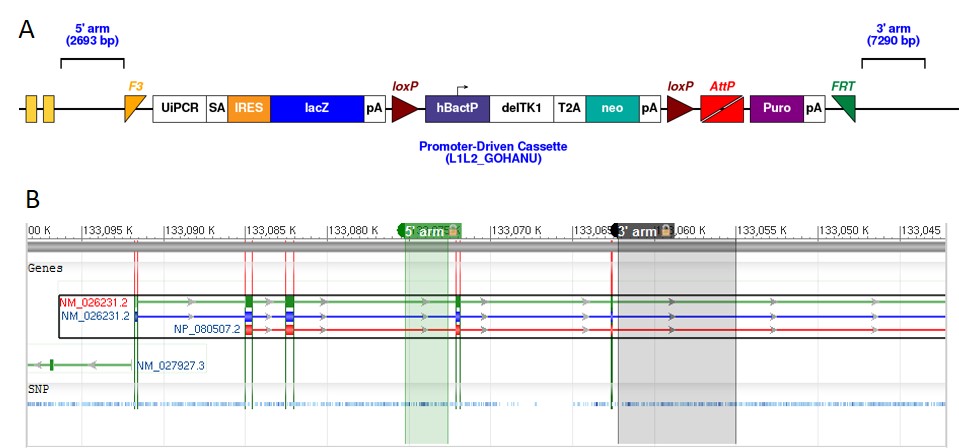

Supplement: S1 Fig — (A) The Promoter driven cassette used to produce the knockout mouse and (B) the region of GSTCD sequences (5’ arm and 3’ arm) inserted into the cassette to make it gene specific on Chromosome 3 GRCm38.p1 C57BL/6J accession number NM_080507. (JPG) [file pone.0221899.s002.jpg]

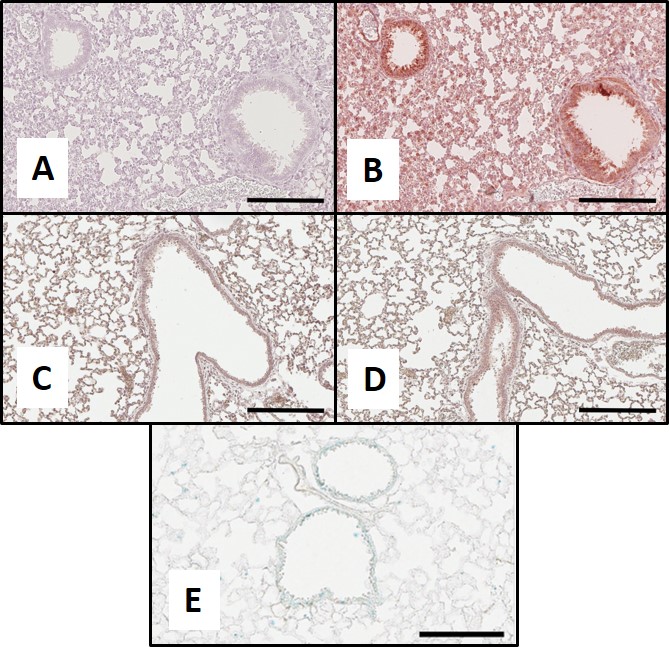

Supplement: S2 Fig — The lung sections show control (no antibody) and stained with anti-GSTCD antibody respectively (A and B) show GSTCD+/+ and (C and D) GSTCD-/- lungs. (E) Xgal staining in GSTCD-/- lung for LacZ expression. Scale bar– 200 μm. (JPG) [file pone.0221899.s003.jpg]
